# Dr. George Frederick Shrady

**Published:** 1908-01

**Authors:** 


### OBITUARY.

Dr. George Frederick Shrady, of New York, died at his home November 29, 1907, aged 70 years. He suffered from pyemia as a sequence of cholelithiasis, his illness being of two weeks duration. Dr. Shrady was a native of New York, where he was born January 14, 1837. His preliminary training was received in the public schools and his academic education at the Free Academy, which afterward became the College of the City of New York, and he received his doctorate degree from the College of physicians and surgeons in 1858, being awarded the Wood intercollegiate prize for proficiency in anatomy. He served as interne in the surgical division of the New York hospital and became an assistant surgeon during the civil war. Near the close of hostilities he resumed practice in New York. He acquired stenography in his earlier years, hence his services became valuable in reporting the meetings of medical societies for which purpose these bodies often employed him.

Dr. Shrady founded the *Medical Record* in 1866, and continued as its editor for thirty-eight years, resigning his position in July, 1904. In addition to his work as editor he was a frequent contributor of papers of a popular character to lay magazines. His contributions to medical literature were many and able, being chiefly of a surgical nature. He was a surgeon first, and then an editor, adorning both positions by his skill and erudition.

Dr. Shrady lent his powerful influence as editor of the *Medical Record* toward freeing the profession of the Empire State from the incubus of the antiquated code of ethics, and fortunately lived to see the full fruition of his hopes and labors in the final abolishment of that useless instrument by the American Medical Association. Besides being a great surgeon and a great editor, Dr. Shrady was a genial gentleman of polished manners, with the kindest of hearts. It was one of the pleasures of the life of the writer to have enjoyed the personal friendship of this charming gentleman.

Dr. Shrady was visiting surgeon to St. Francis's Hospital for twenty years, and was consulting surgeon there for over six years past. He was also consulting surgeon to the Hospital for Ruptured and Crippled, the Columbus Hospital, the Fordham Home for Incurables, the General Memorial Hospital, the Red Cross Hospital, the Vassar Hospital at Poughkeepsie, and the Hackensack Hospital, New Jersey, and was at one time attending surgeon to the Presbyterian Hospital. He was a member of the Medical Societies of the County and of the State of New York, of the New York Academy of Medicine, of the Practitioners' Society of New York, of the New York Pathological Society, of the Harvey Society, of the American Medical Editors' Association, and of the American Academy of Medicine; and of the Metropolitan, the Ardsley, and the Jekyl Island (Ga.) Clubs. He is survived by his wife, three sons, and a daughter.

